# Virtual reality rehabilitation program on executive functions of children with specific learning disorders: a pilot study

**DOI:** 10.3389/fpsyg.2023.1241860

**Published:** 2023-08-10

**Authors:** Valentina Di Giusto, Giulia Purpura, Carla Fulvia Zorzi, Rosanna Blonda, Elena Brazzoli, Paolo Meriggi, Tarjn Reina, Silvia Rezzonico, Roberta Sala, Ivana Olivieri, Anna Cavallini

**Affiliations:** ^1^IRCCS Fondazione Don Carlo Gnocchi, Milan, Italy; ^2^School of Medicine and Surgery, University of Milano-Bicocca, Milan, Italy

**Keywords:** virtual reality, rehabilitation, executive functions, specific learning disorders, children

## Abstract

**Background:**

The application of Virtual Reality (VR) in the field of rehabilitation has been widely studied, because it has already proven to be an effective intervention for a variety of physical and cognitive conditions. Nevertheless, its application in pediatric rehabilitation is more recent. This pilot study aims to examine whether a VR-rehabilitation program may have positive effects on the Executive Functions (EFs) of children with Specific Learning Disorders (SLD).

**Materials and methods:**

Twenty-four children with diagnosis of SLD participated to the study (range 7–11 years) and performed the VR-training across 6 weeks in the CARE Lab, that was designed with appropriate structural measures and ad hoc fittings, to hide the sophisticated technology necessary to allow the child to experience a rehabilitative setting with recreational and semi-immersive features. Children were evaluated across three main time-points: T0, assessment of cognitive level and EFs immediately before the start of the intervention; T1, assessment of EFs immediately after the end of VR intervention; T2, follow-up of EFs after 6 months from the end of the VR intervention. The rehabilitation programs were customized according to clinical needs and/or single patient’s characteristics, proposing different games with variable complexity levels.

**Results:**

Results showed that scores for visual attention, inhibition, flexibility, and planning abilities were significantly higher than before the intervention, and the most part of these ameliorations were maintained after 6 months.

**Conclusion:**

These findings provide important inputs for the development of new innovative rehabilitation interventions for children with SLD that must be founded in ecological and evidence-based approaches.

## 1. Introduction

Virtual reality (VR) refers to a range of technologies, based on computer-generated simulated three-dimensional environments, that are used to provide complex and artificial sensory information, and to create a virtual world to the user with seemingly real images and sounds (Wilson et al., 1997; Afridi et al., 2022). These Information and Communication Technology solutions vary widely in complexity: from easy-to-use interactive videogame consoles originally intended for entertainment purposes, to sophisticated systems specifically developed for health services (Olivieri et al., 2018). In the last decades the application of VR in the field of medicine and rehabilitation has been widely studied. Although the application of virtual reality to rehabilitation is recent, it has already proven to be an effective intervention method for a wide variety of physical and cognitive conditions. Its use has progressively increased among healthcare professionals in order to provide maximum support and to improve quality of care of adult and pediatric patients (Snider et al., 2010; Maggio et al., 2019). For example, for adult post-stroke patients, VR is widely used with the main goal of giving people with disability the same quality of motor, cognitive, and neuropsychological conventional rehabilitation through an adaptable, multi-faceted and more motivating rehabilitation technique, tailored on patients’ profiles, and with the possibility to elicit positive responses during repetitive functional exercise (Demeco et al., 2023).

According to this rehabilitative approach, in the virtual environment it is possible to offer models of real-life tasks through controlled experimental situations, by measuring and guiding human behavior with a precision that exceeds the capabilities of physical environments. Through these controlled experiences in a virtual environment, it is possible for the patients to learn new motor skills to be utilized in the “real-world” (Levac et al., 2019).

To date, the application of VR in pediatric neurorehabilitation has been primarily studied in children with cerebral palsy and other types of motor impairment (Snider et al., 2010; Lino et al., 2021; Menici et al., 2021; Goyal et al., 2022); in children or adolescents with complex outcomes due to traumatic brain injury (Shen et al., 2020a; Corti et al., 2022); and in children with neurodevelopmental disorders, such as autism spectrum disorder (Frolli et al., 2022; Zhang et al., 2022), attention-deficit/hyperactivity disorder (Rodrigo-Yanguas et al., 2021; Hong et al., 2022), and learning or intellectual disability (Ahn, 2021; Köse et al., 2022; Maresca et al., 2022). There are limited data on the use of VR in the education and rehabilitation of children with Specific Learning Disorders (SLD). Recently, Maresca and collaborators found that a VR-based rehabilitation program had positive effects on some cognitive domains and learning abilities (word-reading and homophonic writing) in children with SLD (Maresca et al., 2022). Moreover, Köse et al. (2022) investigated the effects of VR on rehabilitation of visual perception skills in children with SLD and suggested that a supervised game-based intervention program is effective in improving the several subskills of visual perception.

Although it is accepted that VR can be used for the rehabilitation of patients with SLD, in which several cognitive and neuropsychological skills are involved, its application is still limited. For example, there are no studies available on the use of VR in children with SLD for the enhancement of Executive Functions (EFs), that are fundamental cognitive domains for the development of learning academic skills and could be impaired in these patients (Livesey et al., 2006; Schmidt et al., 2017; Barbosa et al., 2019; Meiri et al., 2019). There are studies that have already highlighted how EFs-based intervention in children with SLD can be effective in improving some cognitive and mental domains (like flexibility or inhibition) as well as learning skills (like fluency or reading decoding skills) (Cartwright et al., 2019; Capodieci et al., 2022); however, the application of VR for this purpose has yet to be considered.

As a matter of fact, EFs are a set of general-purpose control mechanisms, that regulate the dynamics of human cognition and action and represent a core component in development of self-regulation abilities, necessary for carrying out everyday activities (Miyake and Friedman, 2012). Typically, EFs have a key-role both in language, psychomotor and social abilities development, and in learning and academic achievement. Recently, Ernst et al. (2022) confirmed that the relation between EF and math skills already in the preschool period are robust, while other authors highlighted as EFs, in particular cognitive flexibility, are important contributors to academic outcomes and predict academic performance in older children, both about literacy and mathematics achievements (Magalhães et al., 2020).

Thus, this pilot study aims at assessing whether a VR-based rehabilitation program could improve EFs in children with SLD. In particular, in this study, improvement in some EFs, fundamental for learning processes, was assessed after 6 weeks of VR-based intervention. The goal of this exploratory study is to set the bases for the use of a VR in clinical practice; and it could be the starting point for the development of randomized controlled trials to assess the efficacy of this novel method in improving neuropsychological and academic skills of children with SLD.

## 2. Materials and methods

### 2.1. Participants

Twenty-four children were included in this study (19 M, 5 F; mean age 8.9 years), recruited from the Division of Child and Adolescent Neuropsychiatry of the IRCCS Don Gnocchi Foundation—Santa Maria Nascente of Milan (Italy). Only children whose parents/legal guardians signed informed consent were included in this study. Participants were selected according to the convenience sampling method. Ethical approval was waived by the local Ethics Committees because all the procedures being performed were part of the routine care. The study was carried out in accordance with the principles of the Declaration of Helsinki.

For the recruitment, the following inclusion criteria were considered: (i) diagnosis of SLD assessed by a multidisciplinary team according to DSM-5 criteria (American Psychiatric Association [APA], 2013) and to the Italian Guidelines for SLD diagnosis (Istituto Superiore Sanità, 2022); (ii) age between 7 and 11 years; and (iii) intellectual quotient >70 at cognitive evaluation by Wechsler Intelligence Scale for Children-IV (WISC-IV). Exclusion criteria were as follows: (i) children with other genetic, neurological, sensory, or psychiatric conditions; (ii) children with epilepsy or seizures controlled by pharmacotherapy; (iii) children who do not speak the Italian language; and (iv) children with SLD in comorbidity with attention-deficit/hyperactivity disorder (ADHD) or autism spectrum disorder (ASD). The main characteristics of the sample are shown in Table 1.

**TABLE 1:** Principal characteristics of participants.

Patients	Gender (M/F)	Age	Total IQ	Type of learning disorder*	Comorbidity**	6-month follow-up
1	M	8	105	DL + DG	No	Yes
2	M	10	105	DL + DO + DG + DC	No	Yes
3	M	9	90	DL + DG	No	Yes
4	M	10	110	DL	No	Yes
5	M	9	90	DO + DG	No	Yes
6	M	10	88	DL + DG	No	Yes
7	M	7	106	DG	DCD	Yes
8	M	11	106	DO + DG	No	Yes
9	F	11	107	DL + DO + DG + DC	No	Yes
10	M	8	97	DL + DO	No	Yes
11	F	8	90	DL + DG	No	Yes
12	M	10	93	DL + DO + DG + DC	No	No
13	M	8	102	DG	DCD	No
14	M	9	103	DL + DO + DG + DC	No	No
15	M	9	90	DG	DCD	No
16	F	10	93	DL + DO + DG + DC	No	No
17	M	7	103	DO + DG	No	No
18	F	7	89	DL + DO + DG + DC	No	Yes
19	M	10	107	DL + DO + DG + DC	No	No
20	M	9	84	DL	No	No
21	M	7	84	DL + DO	No	Yes
22	M	10	73	DL + DO + DG	No	No
23	F	9	84	DL	No	No
24	M	8	101	DL + DG	DCD	Yes

*DL, dyslexia; DO, dysorthography; DG, dysgraphia; DC, dyscalculia. **DCD, developmental coordination disorder.

### 2.2. Procedures

The study was carried out in the CARE Lab of the IRCCS Don Gnocchi Foundation of Milan across three main time-points. T0: cognitive level and EFs were assessed according to a specific protocol (see section “2.2.1. Outcome measures”) and, immediately after the evaluation, recruited children started the VR intervention; T1: at the end of VR intervention (see section “2.2.3. Training procedure”), EFs protocol was administered again; T2: after 6 months from the end of the VR intervention, a follow-up of EFs was performed. All children were evaluated at T0 and T1, but only 14 patients of the original sample completed the follow up at T2. During the VR intervention, participants didn’t attend other treatment.

#### 2.2.1. Outcome measures

The assessment of EFs was performed through a specific protocol created *ad hoc* by the neuropsychiatry team of the CARE Lab. The protocol aims to evaluate three principal functions (visual attention, inhibition, planning and flexibility) using standardized tests. To analyze the scores at the different subtest, authors used the raw or standard scores, the percentile scores, or the scaled scores. In particular, for subtests of NEPSY-II (see below) scaled scores are based on the transformation of raw scores of several subtests in age-appropriate scaled scores (Mean = 10, SD = 3), ranging from 1 to 19.

##### 2.2.1.1. Assessment of visual attention

For the assessment of Visual Attention, the Visual Attention Subtest of the Italian Version of Developmental Neuropsychological Assessment- Second Edition (NEPSY-II) was utilized (Urgesi et al., 2011). This subtest is a time trial of barrage, that evaluates each child’s speed and accuracy in focusing on visual target stimuli (faces with specific characteristics) when they are presented with distractor stimuli. Each child scans an array of pictures and marks the targets as quickly and accurately as possible. For the analysis, a Scaled Score calculated by the number of correct answers (minus errors) was used.

##### 2.2.1.2. Assessment of inhibition

The Inhibition Subtest from NEPSY-II (Urgesi et al., 2011) was also used. This subtest requires the child to look at a series of black and white shapes or arrows and name either the shape, direction, or an alternate response, depending on the color of the shape or arrow. For the purpose of this study, Parts A and B of the test were administered (each one of 80 items): Part A (Naming Condition) requires participants to name the shape of squares and circles, or the up or down direction of arrows; Part B (Inhibition Condition) requires participants to provide the opposite naming response on the same stimuli (e.g., if the child is shown a circle, s/he must say “square”; if s/he sees a square, s/he must say “circle”). For the analysis, the Scaled Scores obtained from time parameter and the Percentile Scores for accuracy parameter were considered.

Moreover, the “Ranette” (in English “Small Frogs”) Subtest of the Italian Battery of ADHD was included (Marzocchi et al., 2010). This subtest involves sustained attention (the test lasts about 10 min), selective attention (children must detect the target sound), and motor inhibition (children must stop the impulsive response to go ahead). In this task, the errors represent a failed inhibition of the answer to “GO” to the right time (difficulty in controlling inhibitory stimuli). It was considered Percentile Scores, that was calculated by using the number of correct responses in the 20 items presented to the child (range raw scores: 0–20).

##### 2.2.1.3. Assessment of planning and flexibility

For the assessment of Planning and Flexibility skills, two tests were selected. First, the Design Fluency Subtest of NEPSY-II (Urgesi et al., 2011) was used. This subtest is designed to assess a child’s ability to generate unique combinations by connecting up to five dots, presented in two arrays: structured and random. The child draws as many designs as possible in a specified time on each array (time constraints: 60 s for each item). The Scaled Score is obtained by using the number of correct tasks (range raw scores: 0–70). Second, the Mazes subtest of the WISC-III (Wechsler, 1991) was included. This subtest consists in 10 mazes of various sizes and complexity. In each maze the child must draw a line from the center to the outside without crossing any of the lines that indicate walls. All items are timed. The Standard Score is calculated through a ratio between execution time and number of errors allowed (range raw score: 0–28).

#### 2.2.2. VR-apparatus—The CARE lab

**FIGURE 1:**
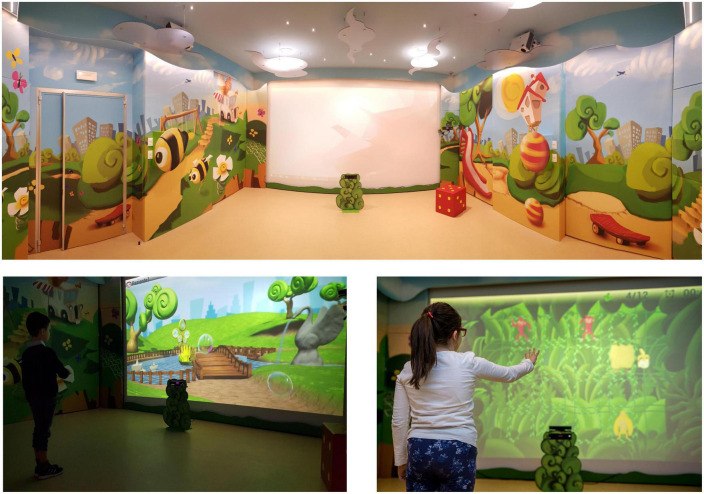
View of the CARE lab. Images of games on VITAMIN reproduced with permission from IRCCS Fondazione Don Gnocchi of Milan (Italy).

The CARE Lab (*C*omputer *A*ssisted *RE*habilitation *Lab*oratory) is a physical space for the study and integration of innovative solutions and their effectiveness in clinical practice, through a multidisciplinary approach. This space was specifically developed by a team of different professionals (rehabilitation specialists—physicians and rehabilitation therapists, engineers and graphic designers), as described in a recent publication (Olivieri et al., 2018). The entire space is designed with appropriate structural measures and “*ad hoc*” fittings, in order to hide the sophisticated technology necessary to allow the child to experience a rehabilitative setting with recreational and semi-immersive features (i.e., screen, sounds appliances), reducing as much as possible the artificiality aspect. Moreover, the CARE lab has been equipped with sensors, in order to record data relating the patients’ movements during rehabilitation sessions (i.e., Kinect–Microsoft–Redmond, WA, USA). The CARE lab has been developed with the aim of support children’s motivation and participation during rehabilitative exercises (see Figure 1). In the context of this laboratory, Olivieri and collaborators have developed a novel software architecture called VITAMIN (VIrtual realiTy plAtform for Motor and cognItive rehabilitatioN), with main features as follows: (i) to collect quantitative information concerning the activities performed by the patients (through sensors like Kinect); (ii) to permit the execution of a personalized set of activities for each patient based on individual abilities; and (iii) to accomplish a “videogame approach” in terms of graphic design, interaction sequences, music and sounds, using a videogame engine (Unreal Engine, Epic Games, Cary, NC, USA). Moreover, VITAMIN used a simple web interface, that can be easily controlled by professional with a tablet; it is a medical device (certified I class), and it provides the access to three different types of games to improve EF, suitably designed by rehabilitation professionals; rehabilitation programs can be customized according to clinical needs and/or single patient’s characteristics, proposing different games with variable complexity levels (task difficulty, stimuli number and size, execution speed, task duration).

#### 2.2.3. Training procedure

Children performed the training with the supervision of a Neurodevelopmental Disorders Therapist or Speech Therapist across 6 weeks (2 weekly session of 45 min).

**FIGURE 2:**
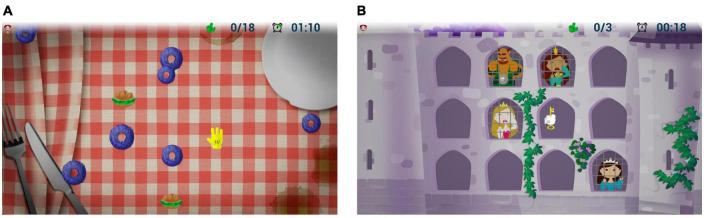
Examples of game activity: **(A)** Gita al Parco: the child must hit the donuts and sandwiches when the distractor appears (for example an ice cream); **(B)** Pronti Via! The child must free the princesses with the key held in the hand (effector), according to the sequence of the appearance of each and ignoring the distractor (ogre). Images of games on VITAMIN reproduced with permission from IRCCS Fondazione Don Gnocchi of Milan (Italy).

We have selected two out of the three available games on VITAMIN (see Figure 2), as follows:

1. “Gita al parco”—(Italian for “Trip to the park”): it provides moving targets and distractors on the screen, which the subject must reach with the upper limb or avoid them. It is used for enhancing visual attention, inhibitory control and shifting, as well as the upper limb quality movements (adduction-abduction in frontal plane).
2. “Pronti, via!”—(Italian for “Ready, go!”): in this game, a mixed sequence of targets (from a minimum of 2 to a maximum of 5), with some distractor (from a minimum of 0 to a maximum of 2) is proposed to the child on the screen. He/she must memorize the sequence and hit the targets by a specific movement of flexion-extension of the arm to reproduce the sequence in exact or reverse order. It is used to reinforce EFs like visual attention and working memory, as well as upper limb control movements.

The game activities have been designed to gradually increase difficulty level within every task (in order to stimulate a progressive enhancement of different functions—based on “zone of proximal development” theory); planning abilities are also emphasized, because the targets tasks move in a random way and with progressively higher speed, so the child must quickly decide the shortest way to reach them avoiding the distractors, or choosing the right strategy to hit as many targets as possible.

During a typical rehabilitation session, the therapist records each child’s demographic information (name, age, sex), laterality, and game parameters (such as virtual targets speed, game’s length, selected limb for the activities etc.). The therapist then starts the calibration procedure by asking the child, who is standing in a fixed position on the floor, to move the arm in the frontal plane as s/he was drawing circles as large as possible. This procedure is essential in order to determine the limb working volume to be selected for the session. As the stimuli are automatically projected onto the screen and transmitted through the loudspeakers by the software, the therapist can concentrate on the child’s activities, while data (such as recording times or performance information) are captured by the sensing devices. At the end of each session, overall scores, number of targets hit, number of errors, type of movements, and other results are reported on the tablet. During game activities, the child’s movements acquired by sensing devices (i.e., Kinect) are translated into digital information to determine the audio/video feedback to present to the user; these data are then stored into specific databases for further analysis after the rehabilitation intervention.

#### 2.2.4. Statistical analysis

For the statistical analysis, the mean values and standard deviation of clinical scores at the different outcome measures were determined by using IBM^®^ SPSS^®^ Statistics software (IBM SPSS Statistics Version 28.0. Armonk, NY, USA: IBM Corp). After evaluating the normal distribution of the dataset for each measure by using Shapiro-Wilk’s Test, Wilcoxon Test or paired-sample Student’s *T*-test were used to determine the presence of significant differences in different parameters across different time-points (T0 vs. T1; T1 vs. T2; T0 vs. T2). The statistical level of significance was set by the *p*-value (*p* ≤ 0.05). Finally, only for the statistically significant comparisons, we calculated the effect size. For the Student’s *T*-test, effect size was calculated by Cohen’s d (small effect, *d* = 0.2; medium effect, *d* = 0.5; large effect, *d* ≥ 0.8), while for the Wilcoxon Test, effect size was given by the matched rank biserial correlation (values range from −1 complete dominance of the second sample, so all values of the second sample are larger than all the values of the first sample, to + 1 complete dominance of the first sample, so all values of the second sample are smaller than all the values of the first sample).

## 3. Results

### 3.1. Visual attention

**FIGURE 3:**
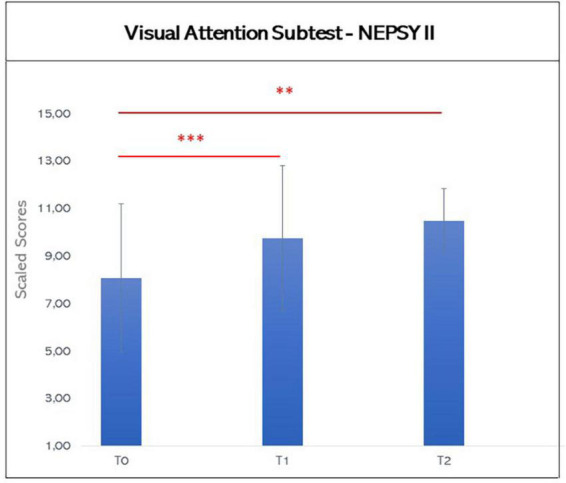
Graphic representation of mean scores in the Visual attention subtest of NEPSY-II at T0, T1, and T2. The asterisks indicate significant difference (*p ≤ 0.05; **p ≤ 0.01; ***p ≤ 0.005). Error bars indicate standard deviations.

Based on the results at Shapiro-Wilk’s Test, it was decided to use a non-parametric approach for analyzing the scores before and after the VR-training. The Wilcoxon Test showed a statistical difference in Visual Attention Subtest between T0 and T1 and between T0 and T2 (T0: mean 8.08, SD: 3.13; T1: mean 9.75, SD 3.05; T2: mean 10.50, SD 1.34; T0 vs. T1: *z* = −3.361, *p* < 0.001, effect size from T1 to T0 *r* = 0.956; T0 vs. T2: *z* = −2.756, *p* = 0.006, effect size from T2 to T0 *r* = 0.939), but not between T1 and T2 (T1 vs. T2: *z* = −1.362, *p* = 0.190), suggesting an improvement in visual attention abilities after the training, that was maintained over the time (see Figure 3).

### 3.2. Inhibition

**FIGURE 4:**
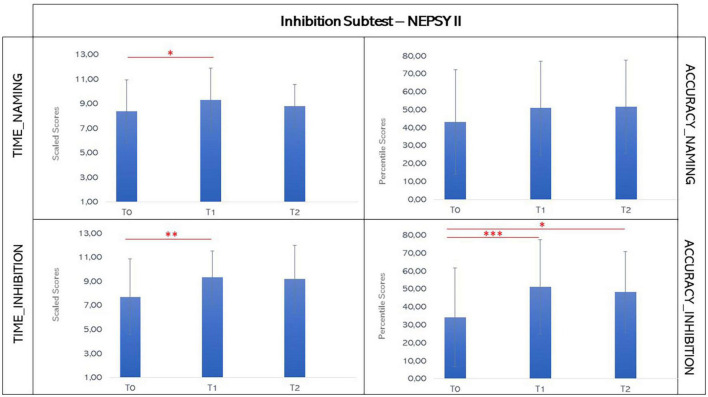
Graphic representation of mean scores in the inhibition subtest t of NEPSY-II at T0, T1, and T2. The asterisks indicate significant difference (*p ≤ 0.05; **p ≤ 0.01; ***p ≤ 0.005). Error bars indicate standard deviations.

**FIGURE 5:**
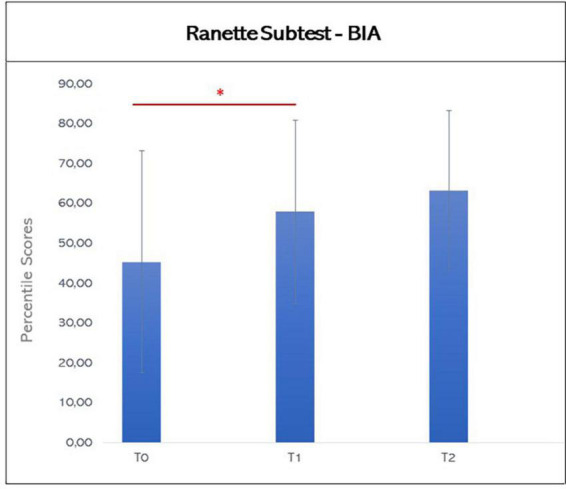
Graphic representation of mean scores in the Ranette subtest of BIA at T0, T1, and T2. The asterisks indicate significant differences (*p ≤ 0.05; **p ≤ 0.01; ***p ≤ 0.005). Error bars indicate standard deviations.

For the analysis of the Scores of the Parts A (Naming) and B (Inhibition) of Inhibition Subtest, the parameters “time” and “accuracy” were considered. For the first parameter, Shapiro-Wilk’s Test showed a normal distribution of data, suggesting the use of a parametric methodology; while for the second, Shapiro-Wilk’s Test revealed a non-normal distribution of data, thus a non-parametric approach was utilized. Also, for the “Ranette” Subtest, the presence of a normal distribution of data, indicated the use of parametric statistics (see Figures 4, 5).

Regarding the Naming Condition (Part A of the Inhibition Subtest), the paired-sample Student’s *t*-test showed an increase of Scaled Scores of the time parameter between T0 and T1, but not between T0 and T2 (T0: mean 8.37, SD: 2.55; T1: mean 9.33, SD 2.59; T2: mean 8.79, SD 1.76; T0 vs. T1: *t* = −2.534, *p* = 0.019, effect size from T1 to T0 0.517; T0 vs. T2: *t* = −0.494, *p* = 0.629). Moreover, it was evident a decrement of the Scaled Scores after the 6 months from the end of the training, with a tendency to significance between T1 and T2 (T1 vs. T2: *t* = 2.144, *p* = 0.052, effect size −0.573), suggesting that the improvement amelioration was not stable at the 6-month follow-up. No statistical differences between the three time-points were found about the accuracy of Naming Condition through Wilcoxon Test (T0: mean 43.25, SD: 29.1; T1: mean 51.04, SD 26.1; T2: mean 51.7, SD 25.9; T0 vs. T1: *z* = −1.221, *p* = 0.232; T0 vs. T2: *z* = −1.423, *p* = 0.168; T1 vs. T2: *z* = −1.183, *p* = 0.265).

By contrast, for Inhibition Condition (Part B) the paired-sample Student’s *t*-test showed an enhancement in the Scaled Scores of the time parameter between T0 and T1 (T0: mean 7.71, SD: 3.15; T1: mean 9.33, SD 2.2; T0 vs. T1: *t* = −2.985, *p* = 0.007, effect size from T1 to T0 0.609), that was stable at 6-month follow up (T2: mean 9.21, SD: 2.78; T1 vs. T2: *t* = 0.162, *p* = 0.874; T0 vs. T2: *t* = −1.298, *p* = 0.217). With respect to the accuracy, an improvement of Percentile Scores was apparent through Wilcoxon Test at the end of the VR-training, with a significant difference between T0 and T1 and T0 and T2, although a slight decrease of accuracy was present at the 6-month follow-up (T0: mean 34.3, SD: 27.4; T1: mean 51.3, SD 26.2; T2: mean 48.3, SD 22.6; T0 vs. T1: z = −3.051, *p* = 0.002, effect size from T1 to T0 *r* = 0.868; T0 vs. T2: *z* = −2.040, *p* = 0.045, effect size from T2 to T0 *r* = 0.667; T1 vs. T2: *z* = −0.524, *p* = 0.675).

Similarly, paired-sample Student’s *t*-test found an improvement at the score of “Ranette” Subtest between T0 and T1 (T0: mean 45.33, SD: 27.8; T1: mean 57.9, SD 23.1; T0 vs. T1: *t* = −2.544, *p* = 0.018, effect size from T1 to T0 0.519), that was maintained at 6-month follow up (T2: mean 63.21, SD: 20.15; T1 vs. T2: *t* = −1.235, *p* = 0.239; T0 vs. T2: *t* = −1.835, *p* = 0.089).

### 3.3. Planning and flexibility

**FIGURE 6:**
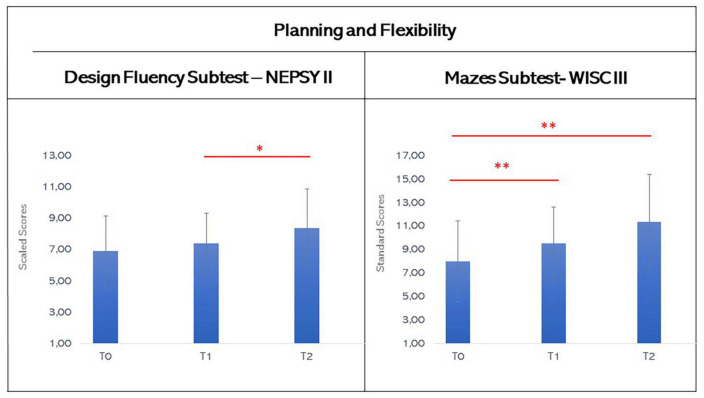
Graphic representation of mean scores in the design fluency subtest of NEPSY-II and in the Mazes subtest of WISC-III at T0, T1, and T2. The asterisks indicate significant differences (*p ≤ 0.05; **p ≤ 0.01; ***p ≤ 0.005). Error bars indicate standard deviations.

For the analyses of these subtests, a non-parametric statistic was used since the Shapiro-Wilks’s Test showed a non-normal distribution of data. In regard to the Design Fluency Subtest’s scores (see Figure 6), Wilcoxon test found a not statistically significant difference between T0 and T1 (T0: mean 6.9, SD: 2.25; T1: mean 7.4, SD 1.9; T0 vs. T1: *z* = −1.215, *p* = 0.231), while there was a statistical significance between T1 and T2 (T2: mean 8.36, SD 2.5; T1 vs. T2: *z* = −2.191, *p* = 0.030, effect size from T2 to T1 *r* = 0.782, T0 vs. T2: *z* = −1.185, *p* = 0.258).

Finally, Wilcoxon Test highlighted a statistical difference in Mazes Subtest between T0 and T1 and T0 and T2, but not between T1 and T2 (T0: mean 8.00, SD: 3.43; T1: mean 9.50, SD 3.13; T2: mean 11.36, SD 4.03; T0 vs. T1: *z* = −2.765, *p* = 0.006, effect size from T1 to T0 *r* = 0.743; T0 vs. T2: *z* = −2.401, *p* = 0.018, effect size from T2 to T0 *r* = 0.818; T1 vs. T2: *z* = −1.172, *p* = 0.258), suggesting an amelioration of planning abilities and of mental flexibility after the VR-training, that was maintained over the time (see Figure 6).

## 4. Discussion

Previous research hypothesized that VR could be employed to improve some cognitive domains of children with developmental disabilities (Shen et al., 2020b; Ahn, 2021; Zhao et al., 2022); however, to the authors’ knowledge this is the first study that provides information on the possible effects of a VR-based rehabilitation program on EFs in children with SLD. The results obtained in this study confirmed the hypothesis that a neuropsychological intervention based on VR methodology could improve EFs in children with SLD.

In fact, this study’s results showed that, after 6 weeks of rehabilitation, the scores for visual attention, inhibition, flexibility, and planning abilities were significantly higher than before the intervention in affected children; and that for the most part these improvements were maintained after 6 months. Based on these data, VR training can be considered a valid rehabilitation approach to use either alone or in combination with conventional rehabilitation methods in children with SLD, in whom EFs are known to be impaired. This is in line with results recently obtained by Shen et al. (2020b), who tested a VR-based system for EFs rehabilitation in children with traumatic brain injury.

Moreover, this type of intervention takes into consideration the multiple deficit model (MDM) for understanding SLD, since that learning requires complex interactions between different specific processes and general cognitive abilities. According to this theoretical framework, multiple etiological factors (genetic and environmental) produce the behavioral symptoms of several developmental disorders by influencing the overall development of relevant neural systems and cognitive processes (van Bergen et al., 2014). For these reasons, a rehabilitative approach that don’t focus primarily on the specific learning processes, but on the EF components, could be a good strategy for this population.

An advantage of using VR for neuropsychological rehabilitation is that children with SLD are free from the direct demands that are often stressing for them, in which intellectual abilities are intact while learnings and academic skills appear impaired. In contrast to traditional social environments, VR-based intervention can provide immediate and predictable responses in a more motivating context. This aspect was widely supported in this study, as shown by the positive feedback received from both the study’s participants and their parents. Moreover, the feasibility of this approach is suggested also by the fact that all participants successfully completed the 100% of training sessions.

Embracing the embodied cognition concept, the VR approach involves the patient’s body to control game, reproducing in an ecological and more appealing way the problems that children face during daily life. According to this idea, the use of VR in neuropsychological rehabilitation confirms that the mind and body are inextricably linked, and that children learn through performing actions and experiencing their consequences in relation to cognitive constructs (Smith and Gasser, 2005). According to Gallese and Lakoff (2005), rational thought is an exploitation of the normal operations of our bodies, and from these processes also derive the construction of human language, that makes use of concepts of which we make experiences. As such, this process is largely unconscious. Through VR-programs, perceptual and motor systems as well as language and cognitive abilities are involved in a synchronized manner to work on specific tasks.

Another advantage of the use of VR in pediatric rehabilitation of EFs is that it provides a safe environment for participants to engage in training, with the possibility of generalizing learnings to different fields and scenarios. For example, the possibility to provide explicit feedback about performance and results can guide the child through the process of monitoring and modifying their own actions, in order to help him/her develop a more efficient behavior in several environments. According to this idea, the skill’s transfer becomes the key features of this rehabilitation approach, because skills’ acquisition occurs in a context that differs from the environment in which the skill is to be expressed. This aspect is very important for children with SLD, because it may have positive cascading effects on their approach to academic tasks. This is supported also by Capodieci et al. (2022), who suggested that computerized activities aimed at implementing cognitive processes in a more general way. Therefore, about exercises on EFs, it is possible to provide more generalized improvements compared to exclusively specific activities on instrumental skills.

In conclusion, our results highlighted the feasibility and the usefulness of a VR rehabilitation program on improving EFs in children with SLD; while also showing the need to develop more accurate outcome measures in order to assess its effectiveness.

## 5. Limitations

This study has some limitations, including the lack of a control group with a randomized approach and the exclusive use of outcome measures about EFs, without considering academic skills. In particular, the lack of a control group limited us to studying the efficacy of the VR rehabilitation program on neurodevelopmental outcomes but also it does not permit to perform other more adequate statistic tests, avoiding the increased risk associated with multiple testing. Moreover, the absence of the assessment of academic skills didn’t allow to measure in a more specific way the transfer of ameliorations in EFs on learnings. Nevertheless, the interesting results obtained in normative “usual care” could be the starting point for successively investigating the efficacy of this type of intervention in children with SLD; this is the first study that assessed the possibility to use the VR for the rehabilitation of SLD children, thus it is the authors’ opinion that that the limitations of this study are outweighed by its novelty.

## 6. Conclusion

Executive Functions are crucial for learning and cognition and are often compromised in children with SLD, although global cognitive level is in the range of the norm. This study presents the application of a VR rehabilitation program on EFs for children SLD within the usual routine care in a child rehabilitation service. This model has been shown to be reliable, flexible, and effective for children with SLD, and our data supports its feasibility and the necessity of implementation in future randomized controlled trials. According to our preliminary results, Information and Communication Technology devices and systems may have remarkable and profound influences on the modalities to think rehabilitation processes. These results provide important inputs for the development of new innovative rehabilitation interventions for children with SLD that must founded in ecological and evidence-based approaches. However, further studies are needed to confirm the efficacy of this approach, also considering the different typical biases of the research in the field of rehabilitation.

## Data availability statement

The data analyzed in this study is subject to the following licenses/restrictions: the data are not publicly available due to ethical reasons. The data that support the findings of this study are available from the corresponding author upon request. Requests to access these datasets should be directed to AC, ancavallini@dongnocchi.it.

## Ethics statement

Ethical approval was not required for the study involving human samples in accordance with the local legislation and institutional requirements, because this rehabilitation program is routinely performed in the clinical practice of the Division of Child and Adolescent Neuropsychiatry of the IRCCS Don Gnocchi Foundation Santa Maria Nascente of Milan (Italy). Written informed consent for participation in this study was provided by the participants’ legal guardians/next of kin.

## Author contributions

VD, GP, IO, and AC contributed to conception and design of the study. VD, CZ, RB, TR, SR, and RS collected the data. GP and EB performed the statistical analysis. VD and GP wrote the first draft of the manuscript. All authors contributed to manuscript revision, read, and approved the submitted version.
